# Interpretable Prediction of Interfacial Resistance for an Imidazoline–Sodium Molybdate–Tween 80 Corrosion Inhibitor Using Bayesian-Optimized Stacking

**DOI:** 10.3390/molecules31213978

**Published:** 2026-11-04

**Authors:** Changfeng Fan, Xue Wang, Xinfu Che, Xianghua Zhan, Xiaoli Yin, Debao Gao, Binchao Gao, Qihao Zhao, Yancong Liu

**Affiliations:** 1 Shandong Institute of Petrochemical and Chemical Technology, Dongying 257061, China; 2 Shandong Engineering Research Center for Green Manufacturing and Intelligent Control, Dongying 257061, China; 3 College of Chemical Engineering, China University of Petroleum, Qingdao 266580, China; 4 Jack Artificial Lift Machinery Co., Ltd., Dongying 257061, China; 5 School of Mechanical and Automotive Engineering, Qingdao University of Technology, Qingdao 266580, China; 6 College of Mechanical and Electronic Engineering, China University of Petroleum, Qingdao 266580, China

**Keywords:** corrosion inhibitor, interfacial resistance, Latin hypercube sampling, stacking regression, Bayesian optimization, SHAP

## Abstract

Predicting the performance of multicomponent corrosion inhibitors requires models that relate composition to electrochemical response and provide interpretable formulation guidance. This study combined Latin hypercube sampling and Bayesian-optimized stacking to investigate an imidazoline–sodium molybdate–Tween 80 inhibitor for mild steel in 3.5 wt.% NaCl. A dataset of 90 formulations linked component concentrations to total interfacial resistance, *R*_total_, obtained from electrochemical impedance spectroscopy. The response comprised pore resistance and charge-transfer resistance. The stacking ensemble combined K-nearest neighbors, support vector regression and LightGBM with a ridge regression meta-learner. In ten-fold evaluation following dataset-level hyperparameter selection, stacking achieved R^2^ = 0.9036, a mean squared error of 969.46 (Ω·cm^2^)^2^ and a mean absolute percentage error of 2.22%. SHapley Additive exPlanations (SHAP) assigned normalized mean absolute attributions of 49.7%, 48.0% and 2.3% to Tween 80, imidazoline and sodium molybdate, respectively. These values identify influential predictors within the sampled domain rather than independent chemical contributions. Complementary polarization and surface observations supported adsorption-mediated protection with possible molybdate-associated interfacial reinforcement. By connecting formulation composition, electrochemical response and model attribution, this framework supports the prioritization of inhibitor mixtures under the investigated conditions.

## 1. Introduction

Control of the metal–electrolyte interface is central to corrosion protection through both surface treatments and solution-phase inhibitors. Chemical treatments of AlSi10MnMg and AlMg3 alloys have connected surface preparation with adhesive durability and corrosion behavior [1,2]. Ceramic coatings on laser-textured aluminum and superhydrophobic colored stainless steel provide further examples of protective interfaces engineered through surface morphology and chemistry [3,4]. These approaches establish the broader importance of interfacial protection, while solution-phase inhibitors offer a distinct route to modifying steel surfaces during aqueous exposure.

For mild steel in chloride-containing environments, inhibitor performance depends on molecular structure, solution chemistry and the evolving surface condition. Studies of imidazoline inhibitors have demonstrated distinct responses for general and localized corrosion, as well as sensitivity to molecular hydrolysis [5,6]. Related work on controlled imidazoline release and inhibition beneath surface deposits further illustrates the importance of inhibitor availability and interfacial access [7,8]. These dependencies motivate formulation-specific evaluation under a defined set of solution and exposure conditions.

Imidazoline-based compounds combine nitrogen-containing polar groups with hydrophobic chains, providing a molecular basis for adsorption-mediated protection. Their behavior in mixtures also depends on interactions with inorganic salts and other surface-active components. Wang et al. investigated imidazoline, sodium molybdate and sodium dodecylbenzene sulfonate (SDBS) on mild steel in 3.5 wt.% NaCl [9]. This closely related system provides a useful basis for examining organic–inorganic inhibitor combinations. Concentration-dependent protection has also been reported for benzimidazole–molybdate mixtures in hydrochloric acid and lignosulfonate–molybdate mixtures in carbonated concrete pore solution [10,11]. Together, these studies indicate that molybdate should be considered within the chemistry of the complete formulation and its exposure medium.

Replacing anionic SDBS with nonionic Tween 80 changes the balance of interactions available within the mixture. Research on Tween 80-containing surfactant blends has linked composition to micellization and interfacial tension [12]. A separate study of coco monoethanolamide (CMEA) demonstrated adsorption-mediated inhibition by a nonionic surfactant on mild steel in hydrochloric acid [13]. These findings motivate consideration of surfactants as active interfacial components, although their behavior remains dependent on molecular structure and electrolyte composition. For the present ternary system, the relevant question is how component proportions jointly influence the electrochemical response through changes in adsorption, dispersion and interfacial organization.

Data-driven methods provide a way to organize such composition-dependent responses and guide formulation selection. Winkler et al. combined high-throughput measurements and predictive modeling to screen organic inhibitors for aluminum alloys [14]. Zhao et al. used a small database to guide inhibitor design for magnetic refrigeration materials [15]. Interpretable machine learning has also been applied to maximum corrosion depth to evaluate the contributions of influencing variables [16]. These studies demonstrate the value of linking measured corrosion behavior to suitable predictors. Here, the modeling task concerns component concentrations within one ternary inhibitor family, with resistance from electrochemical impedance spectroscopy (EIS) as the response.

Latin hypercube sampling (LHS) distributes observations across prescribed input ranges through stratification, providing a structured basis for formulation sampling [17,18]. Stacking then combines predictions from different learners through a second regression model, allowing complementary representations of the same composition–response relationship [19]. Bayesian optimization systematically searches the hyperparameter space and has been combined with stacking in engineering prediction problems [20,21]. In the present application, these tools connect sampling and model selection within a single workflow, whose predictive value is assessed against individual regression models.

Interpretability complements predictive accuracy by identifying which formulation variables contribute most strongly to the fitted response. SHapley Additive exPlanations (SHAP) express model predictions as a baseline value plus feature attributions [22]. The resulting explanation depends on the model, the background distribution and the treatment of relationships among predictors [23]. This consideration is especially relevant for mixtures because component concentrations can be constrained by the total dosage. We therefore use SHAP to describe the learned composition–resistance relationship and interpret its chemical implications alongside the electrochemical and surface observations.

This study integrates 90 LHS-based formulations, electrochemical measurements, surface characterization and Bayesian-optimized stacking (BO-Stacking) for an imidazoline–sodium molybdate–Tween 80 inhibitor. The prediction target is total interfacial resistance, *R*_total_ = *R*_p_ + *R*_ct_, obtained from EIS fitting. Model comparisons and residual analysis evaluate predictive performance, while SHAP identifies influential composition variables. The combined analysis establishes a composition–resistance map for formulation prioritization and provides a chemically informed interpretation within the investigated conditions.

## 2. Materials and Methods

### 2.1. Steel Preparation and Imidazoline Synthesis

The chemical composition of the mild steel is listed in Table 1. Samples were cut into sheets measuring 30 × 30 × 1 mm. Before testing, the surfaces were sequentially wet-ground with 240, 500, 800, 1000 and 2000 grit silicon carbide papers. The samples were then rinsed with ethanol and deionized water and dried with a blower.

The imidazoline intermediate was synthesized by reacting a fatty acid with diethylenetriamine. Diethylenetriamine was introduced into a three-necked flask fitted with a phase separator, reflux condenser and thermometer, and heated to approximately 150 °C. The fatty acid and 5 mL of xylene were added under nitrogen. The temperature was increased to 170 °C for acylation and dehydration, then raised stepwise to 210 °C to promote cyclization. Heating was stopped when water collection ceased, and the collected volume reached the theoretical value. Residual diethylenetriamine and xylene were removed under reduced pressure, and vacuum drying yielded a reddish-brown, viscous intermediate.

The intermediate was subsequently heated to 100–120 °C in an oil bath. Benzyl chloride was added, and the reaction was continued for 4 h to obtain a water-soluble imidazoline quaternary salt. This product is designated IM throughout the manuscript.

### 2.2. Formulations and Sampling Design

**Figure 1:**
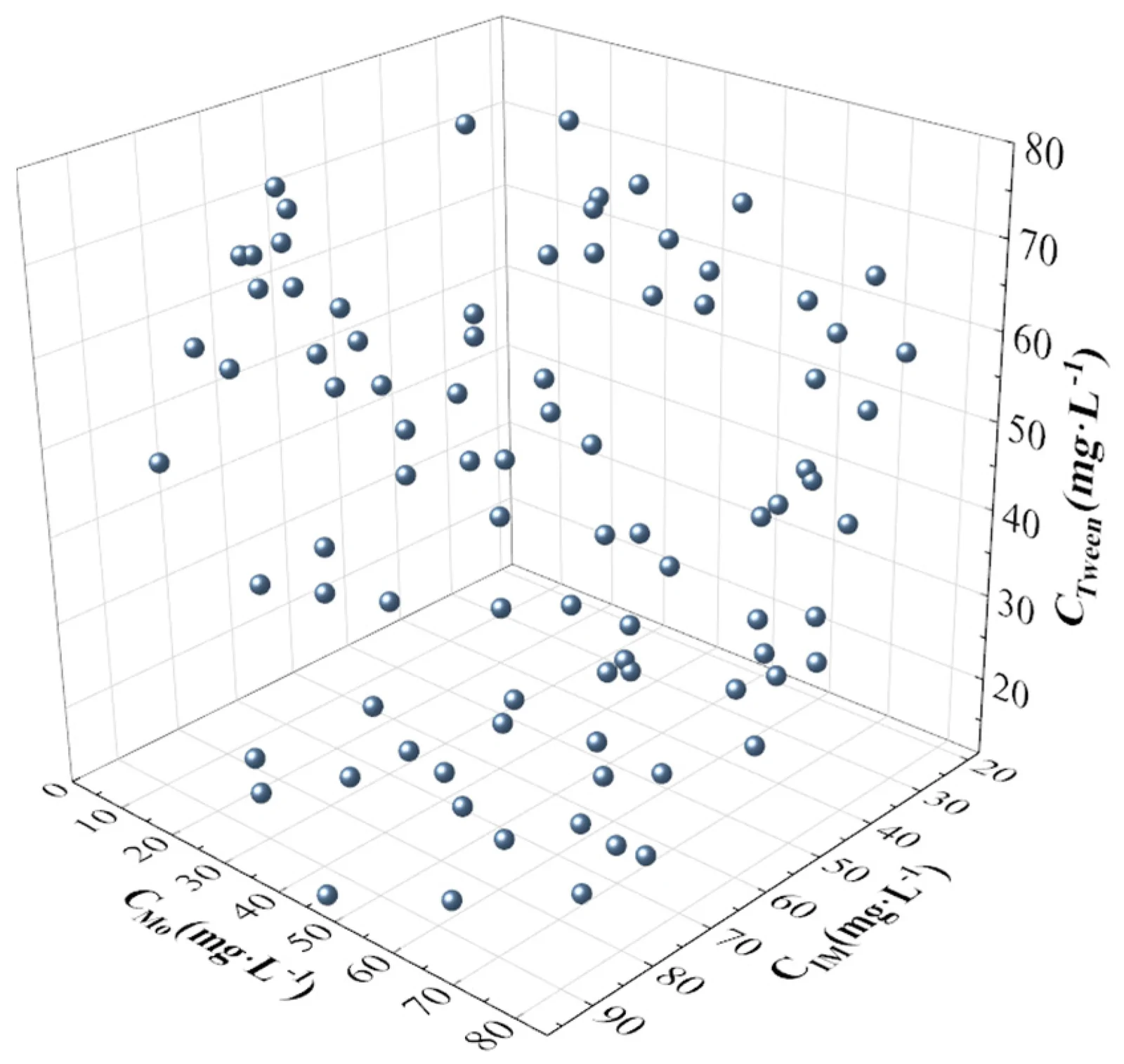
Distribution of the 90 sampled formulations in the concentration coordinates *C*_IM_, *C*_Mo,_ and *C*_Tween_.

The inhibitor comprised IM, sodium molybdate and Tween 80, with concentrations denoted *C*_IM_, *C*_Mo_ and *C*_Tween_, respectively. The formulations were specified at a fixed total inhibitor dosage of 150 mg·L^−1^. An LHS-based design was used to generate 90 concentration combinations [17,18]. *C*_IM_ ranged from 20.00 to 95.00 mg·L^−1^, while both *C*_Mo_ and *C*_Tween_ ranged from 10.00 to 78.00 mg·L^−1^. Figure 1 presents the sampled combinations, and Table 2 summarizes the concentration distributions and measured resistance.

The intended formulation domain was defined by the simultaneous constraints 20 ≤ *C*_IM_ ≤ 95, 10 ≤ *C*_Mo_ ≤ 78, 10 ≤ *C*_Tween_ ≤ 78 and *C*_IM_ + *C*_Mo_ + *C*_Tween_ = 150, with all concentrations expressed in mg L^−1^. These constraints leave two independent composition degrees of freedom. For a selected C_IM_ between 20 and 95 mg L^−1^, the feasible *C*_Mo_ interval is max (10, 72 − *C*_IM_) ≤ *C*_Mo_ ≤ min (78, 140 − *C*_IM_), and the corresponding Tween 80 concentration is determined by *C*_Tween_ = 150 − *C*_IM_ − *C*_Mo_. The conditional bounds on *C*_Mo_ follow from requiring both *C*_Mo_ and *C*_Tween_ to remain within 10–78 mg L^−1^. Thus, the component concentration ranges are coupled by the total-dose constraint and cannot be combined arbitrarily. Increasing one component requires a compensating decrease in at least one other component, so the investigated formulations represent changes in mixture composition at a common total dosage. A row-wise check of the complete modeling dataset confirmed that the recorded component concentrations satisfy *C*_IM_ + *C*_Mo_ + *C*_Tween_ = 150 mg·L^−1^ for all 90 records and fall within the reported component ranges. The fixed-total-dose condition was therefore verified for each individual record.

### 2.3. Electrochemical Measurements and Response Definition

Electrochemical measurements were conducted in 3.5 wt.% NaCl solution using a Gamry Reference 3000 workstation controlled by Echem software, version 5.61. The three-electrode cell comprised a mild-steel working electrode, an Ag/AgCl reference electrode and a platinum-mesh counter electrode. The exposed working-electrode area was 1 cm^2^. Before testing, the open-circuit potential was monitored until a relatively stable value was reached.

EIS measurements were performed at open circuit over a frequency range of 100 kHz to 100 mHz. The 20 mV perturbation amplitude was selected with reference to previous applications in aqueous corrosion-inhibitor studies [24,25]. The spectra were fitted using ZView. Potentiodynamic polarization curves were recorded at 1 mV·s^−1^ over ±0.1 V relative to the open-circuit potential. EIS measurements were conducted in triplicate for each formulation, and each spectrum was fitted separately using the equivalent circuit. For each replicate, the total interfacial resistance was calculated as

*R*_total_ = *R*_p_ + *R*_ct_ (1)

Here, *R*_p_ denotes pore or film-related resistance, *R*_ct_ denotes charge-transfer resistance, and *R*_s_ denotes solution resistance. The arithmetic mean of the three replicate-specific *R*_total_ values was used as the regression target, yielding one response value per formulation record.

Across the 90 formulation records, the formulation-level mean *R*_total_ values ranged from 940.21 to 1358.51 Ω·cm^2^, with an overall mean of 1139.18 Ω·cm^2^ and a standard deviation of 100.84 Ω·cm^2^. These *R*_total_ summary statistics, reported in Table 2, describe variation across the formulation-level mean responses. The standard deviation therefore reflects between-record variation rather than variability among the three repeated measurements within each formulation.

### 2.4. Surface Characterization

Surface morphology was examined by scanning electron microscopy (SEM; SUS3800, Hitachi, Tokyo, Japan). Elemental distributions were characterized by energy-dispersive X-ray spectroscopy (EDS; Xplore30, Oxford Instruments, Oxfordshire, UK). Figures 5 and 6 compare samples immersed for 12 h in the blank and inhibitor-containing solutions. The combined observations provide complementary information on surface damage and the distribution of elements associated with the exposed interface.

### 2.5. Regression Models and Validation

The model comparison included K-nearest neighbors (KNN), support vector regression (SVR), Lasso, CatBoost, LightGBM and a stacking ensemble. Lasso provided a regularized linear reference through coefficient shrinkage [26]. KNN represented local similarity, while SVR with a radial basis function kernel represented smooth nonlinear relationships [27]. CatBoost and LightGBM provided alternative gradient-boosted tree representations [28,29]. The stacking ensemble combined KNN, SVR and LightGBM as base learners, with ridge regression as the meta-learner [19,30]. This structure allowed the meta-learner to combine complementary predictions while regularization constrained the fitted combination coefficients.

Bayesian optimization [20] was used to select hyperparameters before the ten-fold evaluation. The search was conducted on the complete dataset using five-fold cross-validation, 15 iterations, R^2^ as the selection criterion and a random seed of 42. The search covered the number of neighbors for KNN (3–15), C and gamma for SVR (1–100 and 0.001–1, respectively), and the number of estimators for LightGBM and CatBoost (100–300). Gamma was searched on a logarithmic scale. Lasso used a fixed regularization parameter of 10. For ridge regression [30], the regularization parameter was selected over 0.1–100 on a logarithmic scale using the original concentration predictors before incorporation into stacking.

The three component concentrations were used as predictors. StandardScaler was included within the estimator pipelines and fitted on the training subset used for each fit. All models were evaluated using the same shuffled ten-fold partitions with a random seed of 42, while the previously selected hyperparameters remained fixed. The procedure therefore constitutes a non-nested ten-fold evaluation following dataset-level hyperparameter selection. Interpretation of the resulting performance estimates should account for potential model-selection effects [31].

Following the stacked-generalization framework [19], the stacking model used five-fold out-of-fold predictions from KNN, SVR, and LightGBM within each evaluation split to train the ridge meta-learner. The internal partitions were generated with shuffling and a random seed of 42. Each out-of-fold prediction was generated by a base learner fitted without the corresponding internal validation fold. Ridge was trained on the resulting prediction matrix, and the base learners were subsequently refit on the complete training portion to predict the held-out evaluation fold.

Performance was summarized using R^2^, MSE, and MAPE calculated from the pooled held-out predictions, with RMSE obtained as the square root of MSE. Repeated nested cross-validation and confidence intervals were not included in this evaluation. Following model comparison, the final stacking model was refitted using all 90 formulation records for SHAP analysis, separately from the calculation of the evaluation metrics.

### 2.6. SHAP Analysis

Kernel SHAP was used to explain the stacking predictions in terms of *C*_IM_, *C*_Mo_ and *C*_Tween_ [22]. The background distribution was obtained by applying shap.kmeans to the concentration matrix of all 90 formulation records, using 20 representative vectors weighted by cluster membership. SHAP values were calculated for all 90 records with the n samples argument set to 256.

For each feature coalition, retained concentrations were taken from the formulation being explained, whereas masked concentrations were replaced by the corresponding values in the background vectors. The model outputs were averaged over the weighted background distribution. No mixture-specific projection or renormalization was applied to the resulting hybrid inputs. Although individual component values remained within their observed ranges, the hybrid combinations were not constrained to satisfy *C*_IM_ + *C*_Mo_ + *C*_Tween_ = 150 mg L^−1^.

The resulting attributions therefore characterize the fitted model under the specified background and masking procedure. They should not be interpreted as independent component effects or as concentration-substitution effects restricted to the feasible mixture domain [23]. Global importance was summarized by the mean absolute SHAP value for each variable, with its normalized percentage calculated as follows:

(2) $$I_{j}=\frac{100\times \mathrm{mean}(\left|\varphi_{j}\right|)}{\sum_{k}\mathrm{mean}(\left|\varphi_{k}\right|)}$$

where *φ_j_* is the SHAP attribution for variable *j* and the denominator sums the mean absolute attributions over all three concentrations. This quantity measures contribution magnitude across the explained observations. Because the absolute value removes direction, *I_j_* indicates relative model importance rather than whether increasing a concentration raises or lowers *R*_total_.

## 3. Results and Discussion

### 3.1. Electrochemical Behavior and Surface Observations

#### 3.1.1. Potentiodynamic Polarization

**Figure 2:**
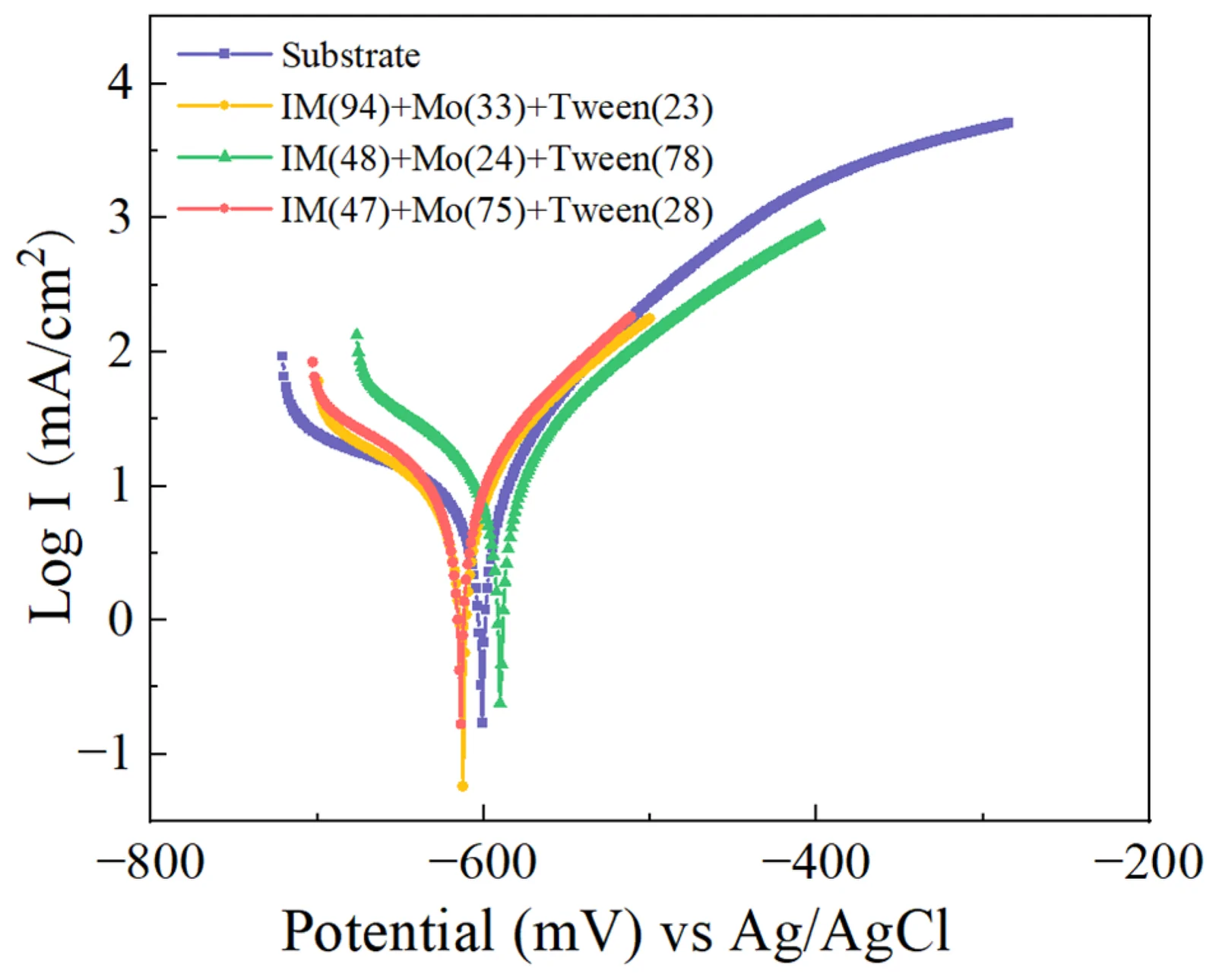
Potentiodynamic polarization curves for mild steel in 3.5 wt.% NaCl without an inhibitor (substrate) and with three representative mixtures. Concentrations in the legend are in mg·L^−1^; each mixture totals 150 mg·L^−1^.

Figure 2 compares the blank solution with three representative mixtures containing *C*_IM_/*C*_Mo_/*C*_Tween_ concentrations of 94/33/23, 48/24/78 and 47/75/28 mg·L^−1^. Each mixture had a total inhibitor dosage of 150 mg·L^−1^. The inhibited samples exhibited lower anodic currents than the blank over much of the displayed anodic region. Differences among the inhibited curves show that redistributing the same total dosage changes the electrochemical response.

The cathodic branches also varied with formulation, although their separation depended on the applied potential. Together, the anodic and cathodic responses are consistent with an inhibitor-modified interface that influences both partial reactions. Changes in surface coverage and access of solution species provide plausible explanations for this behavior. The polarization curves provide a qualitative comparison of the electrochemical responses of the blank and inhibitor-containing solutions. Tafel-derived kinetic parameters are not reported here; therefore, these observations are not used to quantify corrosion rates or inhibition efficiencies. The quantitative response considered in the formulation-prediction analysis is the EIS-derived total interfacial resistance, *R*_total_.

#### 3.1.2. Impedance Response and Equivalent-Circuit Interpretation

**Figure 3:**
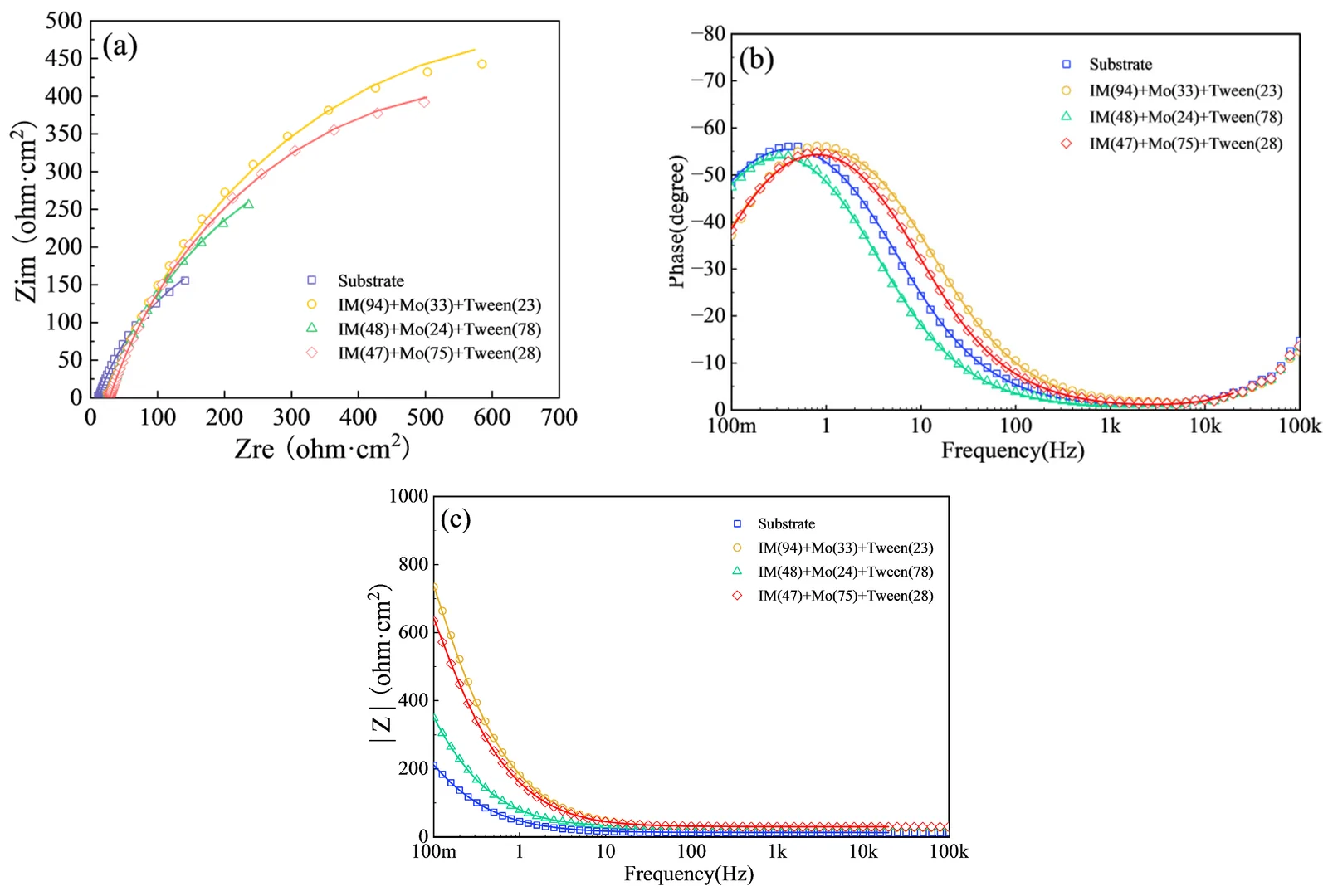
EIS spectra of mild steel in 3.5 wt% NaCl solution without inhibitor and with three representative inhibitor formulations: (**a**) Nyquist plots, (**b**) Bode phase-angle plots and (**c**) Bode impedance modulus plot. Symbols denote experimental data, and solid lines denote equivalent-circuit fits.

Figure 3 presents the Nyquist plots (a), Bode phase-angle plots (b) and Bode impedance modulus plot (c) for the blank and the three representative inhibitor formulations. Symbols represent experimental data, and solid lines represent the corresponding equivalent-circuit fits. The phase-angle plots complement the Nyquist representation by showing the frequency-dependent phase response and facilitating assessment of the relaxation behavior and fitting agreement. Among the illustrated mixtures, the 94/33/23 formulation showed the largest response, followed by 47/75/28 and 48/24/78. This ordering reinforces the composition dependence observed in the polarization curves. The resistance values used for regression were obtained from equivalent-circuit fitting, allowing a consistent comparison beyond the visible portions of the impedance arcs.

**Figure 4:**
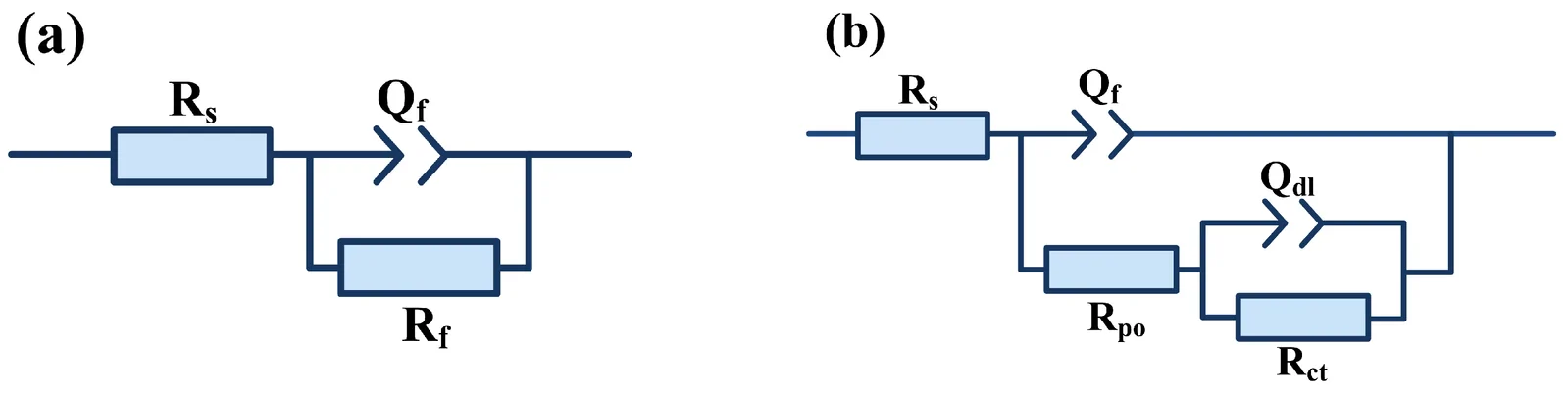
Equivalent-circuit diagrams for (**a**) the blank and (**b**) the inhibited steel. Rs is solution resistance; the inhibited circuit includes pore resistance and charge-transfer resistance.

Figure 4 presents the equivalent circuits used to describe the blank and inhibited responses. The blank circuit comprises solution resistance and one interfacial relaxation branch, whereas the inhibited circuit includes an additional film-related contribution. Nonideal capacitive behavior is represented by a constant phase element (CPE), accommodating the distributed response of a heterogeneous interface. The CPE coefficient is not itself an ideal capacitance; its interpretation depends on the associated circuit parameters [32]. This representation separates the principal resistive contributions while allowing departures from ideal capacitive behavior.

For the inhibited circuit, *R*_total_ combines the film-related and charge-transfer resistances. A larger *R*_total_ indicates stronger opposition to the interfacial processes represented by the circuit, making it a useful descriptor for comparisons under common conditions. Its interpretation remains tied to that circuit definition. Conversion from resistance to corrosion current is a separate step governed by electrochemical kinetic assumptions, as formalized by the Stern–Geary relationship [33].

Using the combined response also reflects the multicomponent nature of the inhibitor. Changes in composition may affect barrier properties and charge-transfer kinetics to different extents while producing similar total resistance values. The regression model captures their aggregate contribution to the measured interface, providing a formulation-level descriptor without assigning the entire response to a single process.

#### 3.1.3. Surface Morphology and Elemental Distributions

**Figure 5:**
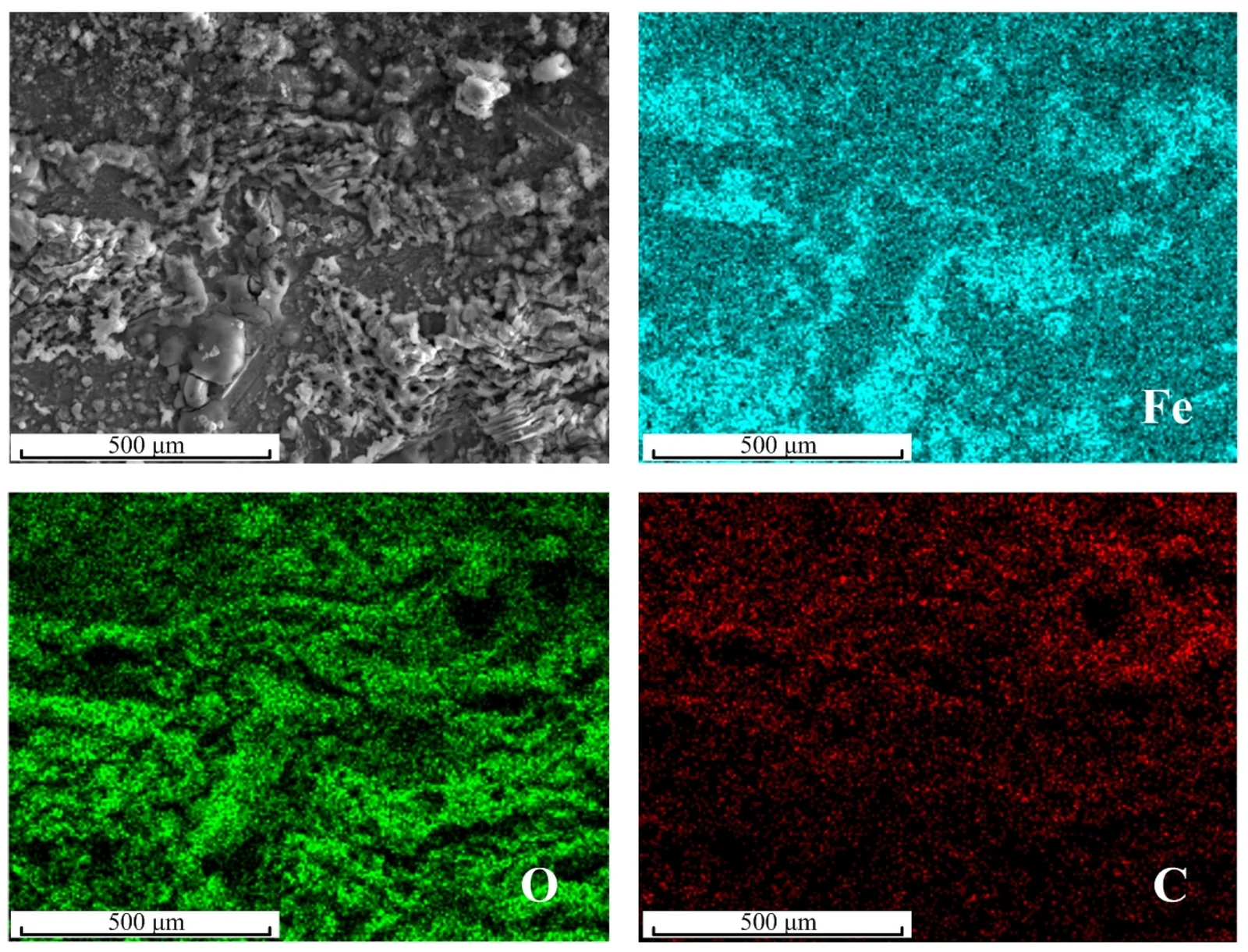
SEM image and EDS maps of Fe, O, and C for the steel sample after 12 h in the blank solution.

**Figure 6:**
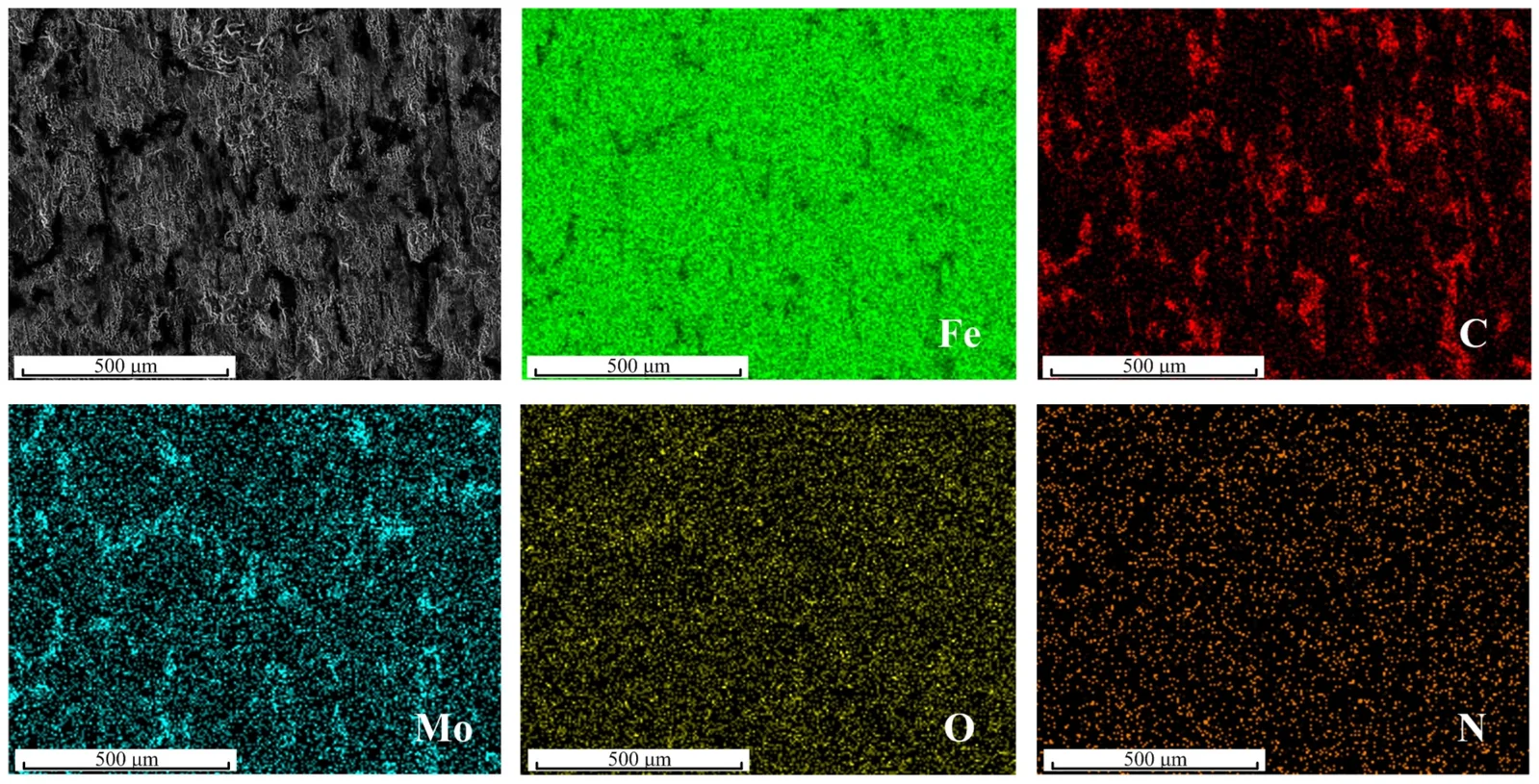
SEM image and EDS elemental maps of the steel sample after 12 h of immersion in an inhibitor-containing solution.

After 12 h of immersion, the blank sample showed a rough surface with extensive deposits and visible corrosion damage (Figure 5). The inhibited sample exhibited a more uniform appearance and less pronounced damage in the observed region (Figure 6). This morphological contrast agrees with the reduced anodic currents and increased impedance in the inhibitor-containing solutions. The three observations therefore provide complementary evidence of improved interfacial protection under the studied conditions.

The elemental maps further distinguish the blank and inhibited surfaces. Fe and O in the blank are consistent with an oxidized iron-containing surface, while Mo in the inhibited sample indicates association of molybdenum-containing material with the interface. Carbon and oxygen provide additional compositional context but do not uniquely identify adsorbed organic molecules. Accordingly, the maps support the presence of inhibitor-associated material, while the proposed chemical roles are interpreted together with the electrochemical response and the relevant literature.

Taken together, polarization, EIS and surface characterization reveal a coherent formulation-dependent protective response. The inhibitor changes interfacial electrochemical behavior and reduces visible surface deterioration over the reported exposure period. These findings provide the experimental context for relating mixture composition to *R*_total_ and for interpreting the model attribution in terms of plausible interfacial processes.

### 3.2. Composition Domain and Variation in Resistance

Across the 90 formulations, *R*_total_ ranged from 940.21 to 1358.51 Ω·cm^2^, corresponding to a span of 418.30 Ω·cm^2^. For reference, the uninhibited blank exhibited an *R*_total_ value of 605 Ω·cm^2^ under the same experimental conditions. The resistance values of the inhibitor-containing systems were therefore approximately 1.55–2.25 times that of the blank, indicating increased interfacial resistance in the presence of the formulations. This comparison provides a quantitative baseline for evaluating their corrosion protection performance and supports formulation screening under the investigated laboratory conditions. The standard deviation corresponded to approximately 8.9% of the mean. This measurable variation provides the response range from which the regression models learn composition-dependent differences. Because all formulations share the same constituent family and nominal total dosage, the comparison focuses on redistribution among the three components.

The fixed-total-dose constraint also gives the concentration coordinates a clear formulation-related meaning. Increasing one ingredient represents substitution for one or both of the others, so the learned relationships describe mixture adjustments rather than isolated additive effects. Figure 1 shows the sampled combinations, and Table 2 summarizes their marginal distributions. Together, these descriptions define the composition domain to which the subsequent model comparisons and SHAP interpretation apply.

### 3.3. Predictive Performance and Model Comparison

**Figure 7:**
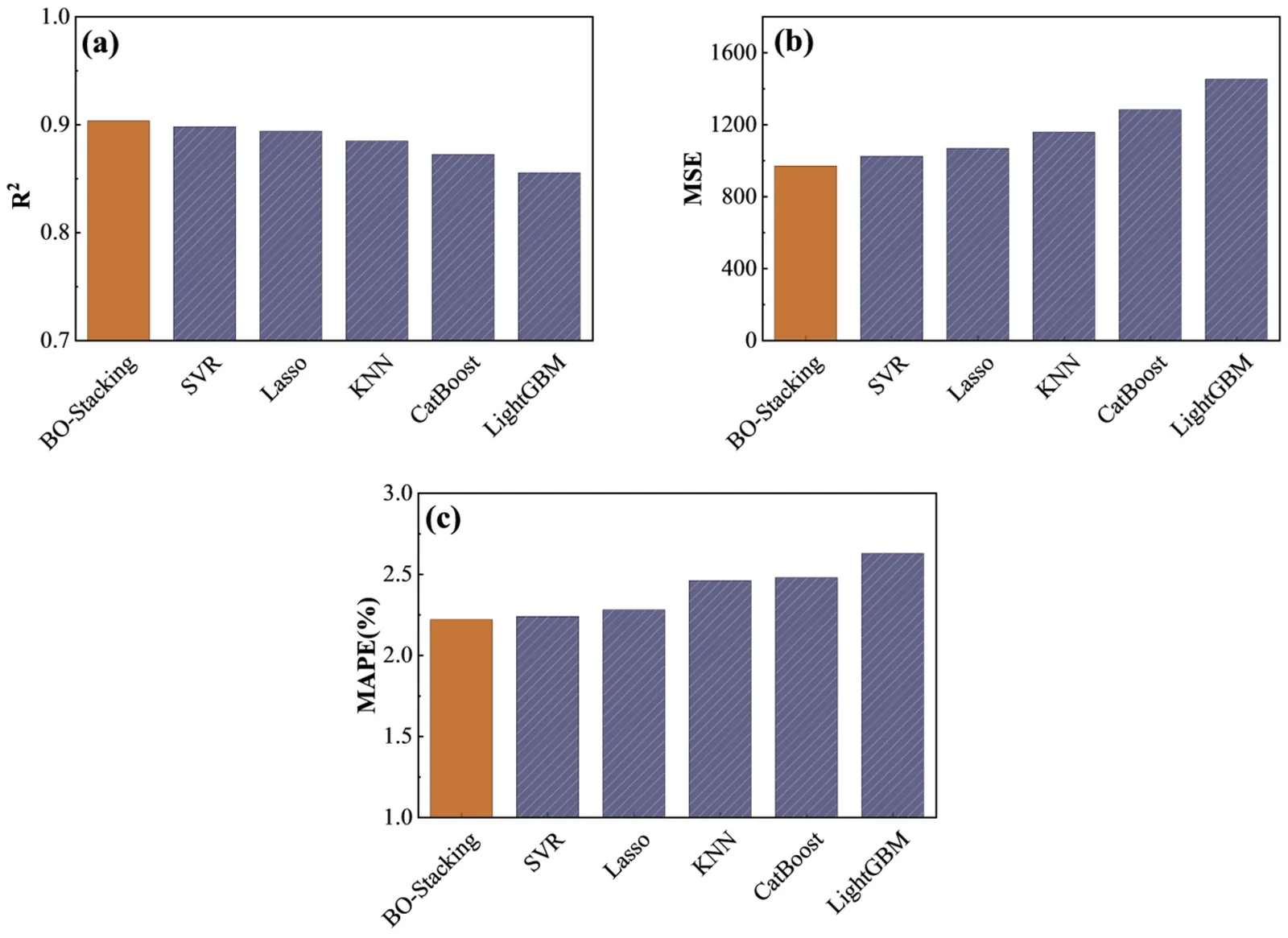
Test performance of the compared regression models: (**a**) *R*^2^, (**b**) MSE and (**c**) MAPE. *R*^2^ is dimensionless, MSE is expressed in (Ω·cm^2^)^2^, and MAPE is expressed as a percentage.

Table 3 and Figure 7 compare the predictive performance of the evaluated models. Stacking achieved the highest test *R*^2^ (0.9036), the lowest test MSE (969.46 (Ω·cm^2^)^2^) and a test MAPE of 2.22%. The corresponding RMSE, calculated from the MSE, was 31.14 Ω·cm^2^. SVR was the strongest individual model, with *R*^2^ = 0.8981, MSE = 1024.43 (Ω·cm^2^)^2^ and MAPE = 2.24%.

Relative to SVR, stacking increased test *R*^2^ by 0.0055 and reduced MSE by 54.97 (Ω·cm^2^)^2^, equivalent to approximately 5.4%. The improvement was incremental, consistent with the close MAPE values of the two models. Stacking therefore offered a favorable combination of the reported metrics, while SVR remained a competitive individual predictor for this dataset.

Lasso also performed well, achieving a test *R*^2^ of 0.8938. Its proximity to the nonlinear models indicates that a regularized linear representation captured much of the resistance variation within the sampled domain [26]. The lower stacking error is consistent with combining complementary local, kernel-based and tree-based predictions [19]. The benefit of the ensemble lies in this combined representation, rather than an assumption that increasing model complexity necessarily improves formulation prediction.

The training–test comparison provides an additional perspective on model selection. CatBoost achieved a training *R*^2^ of 0.9916 but a test *R*^2^ of 0.8724, whereas stacking yielded 0.9300 and 0.9036, respectively. The smaller gap for stacking indicates closer agreement between fitting and predictive performance in this evaluation. These results favor selection based on held-out predictions, rather than the ability to reproduce training observations [31].

### 3.4. Predicted Values and Residual Behavior

**Figure 8:**
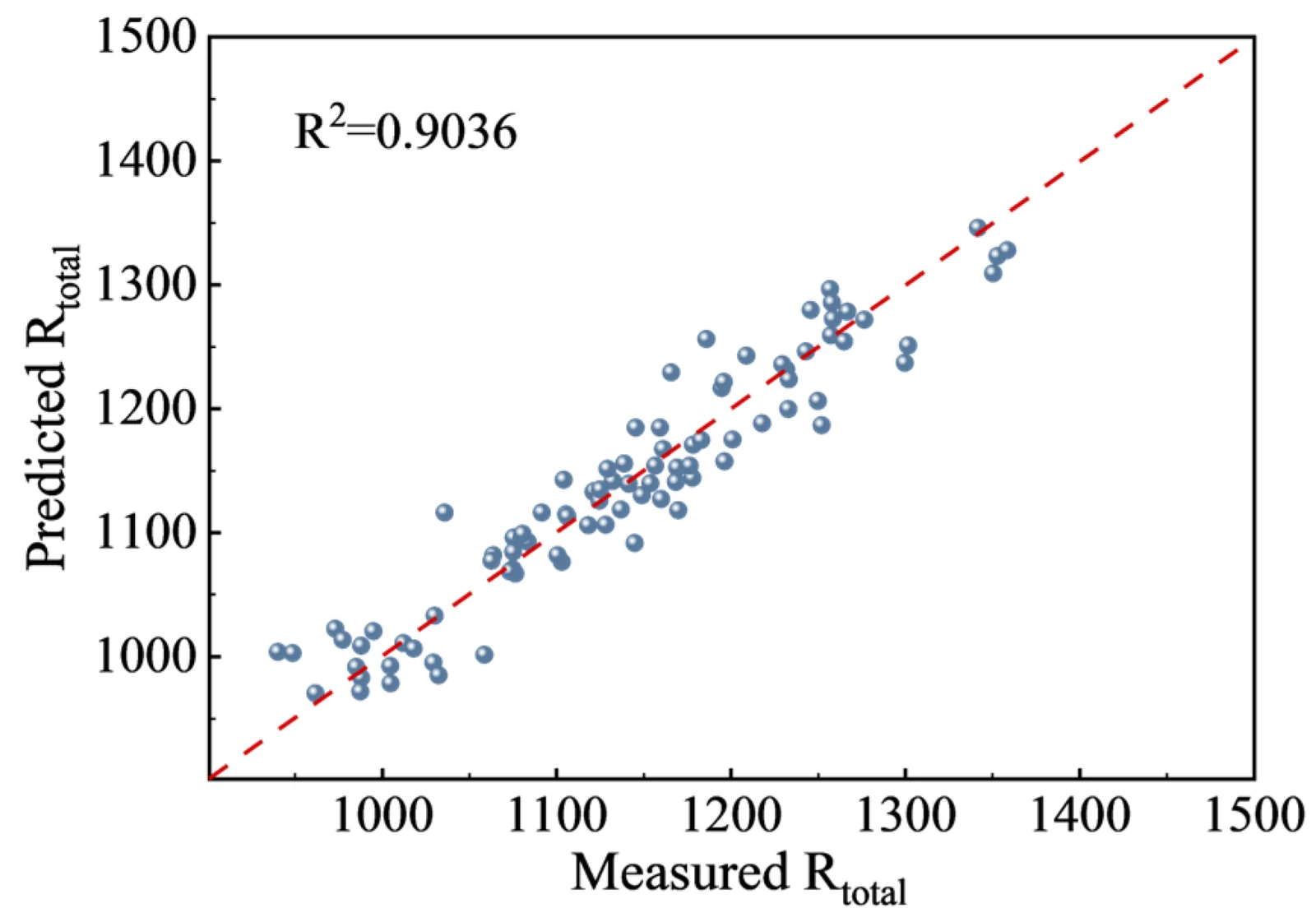
Measured and predicted *R*_total_ values from BO-Stacking outer cross-validation. The dashed line represents equality between predicted and measured resistance.

The BO-Stacking predictions followed the identity line across most of the measured resistance range (Figure 8), capturing the principal differences among formulations. Several observations, particularly toward the upper end of the response range, showed larger deviations. Thus, the model reproduced the overall composition-dependent trend while retaining finite errors for individual mixtures. The parity plot complements the aggregate metrics by showing where those deviations occur across the response range.

**Figure 9:**
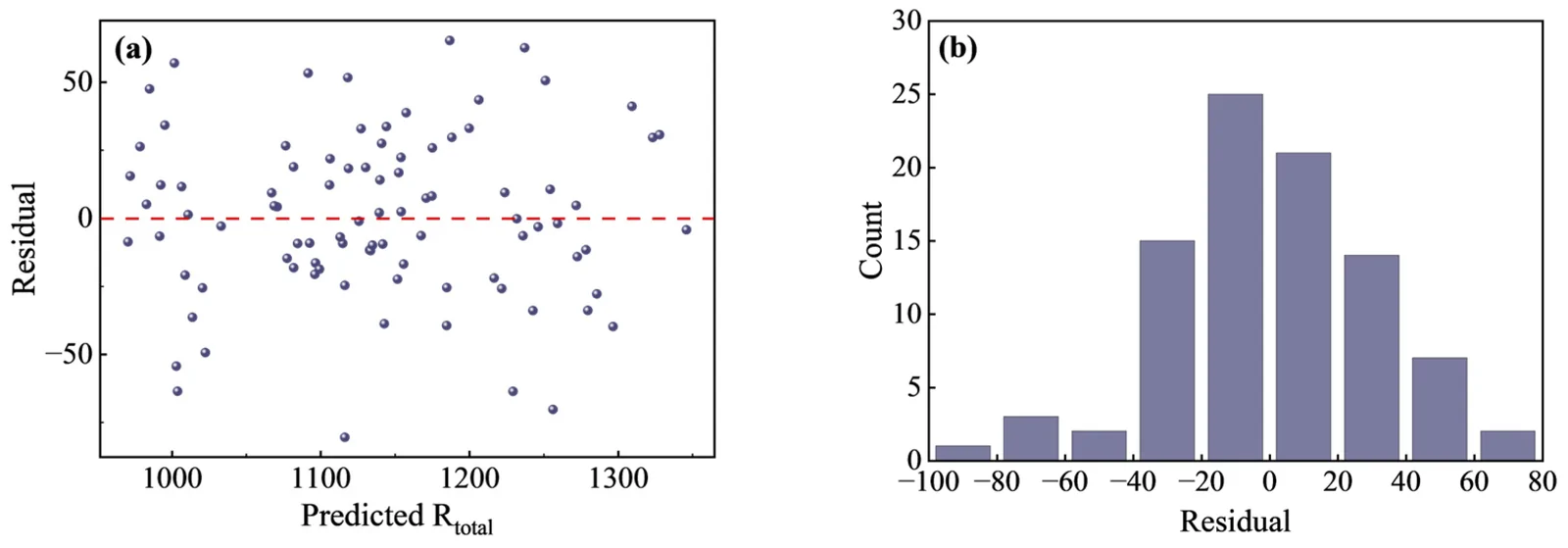
Residual diagnostics for BO-Stacking: (**a**) residuals against predicted resistance, and (**b**) residual frequency distribution. The dashed line in (**a**) denotes zero residual.

Residuals were distributed on both sides of zero across the predicted resistance range, with no obvious monotonic trend on visual inspection (Figure 9a). The histogram was concentrated near zero and contained fewer observations with large deviations (Figure 9b). These patterns are consistent with the model capturing the dominant response trend without a pronounced overall directional bias. The plots provide descriptive diagnostics of predictive errors across the evaluated formulations.

The larger deviations at some high-resistance formulations may reflect local data representation, regression toward the response mean or measurement variability. For formulation prioritization, predicted differences should therefore be considered alongside the overall error scale. Mixtures with nearly identical predicted resistance may be practically indistinguishable at that scale, even when their numerical ranking differs. The model is most useful for organizing composition trends and identifying promising regions of the sampled domain.

### 3.5. Model Attribution and Formulation Effects

**Figure 10:**
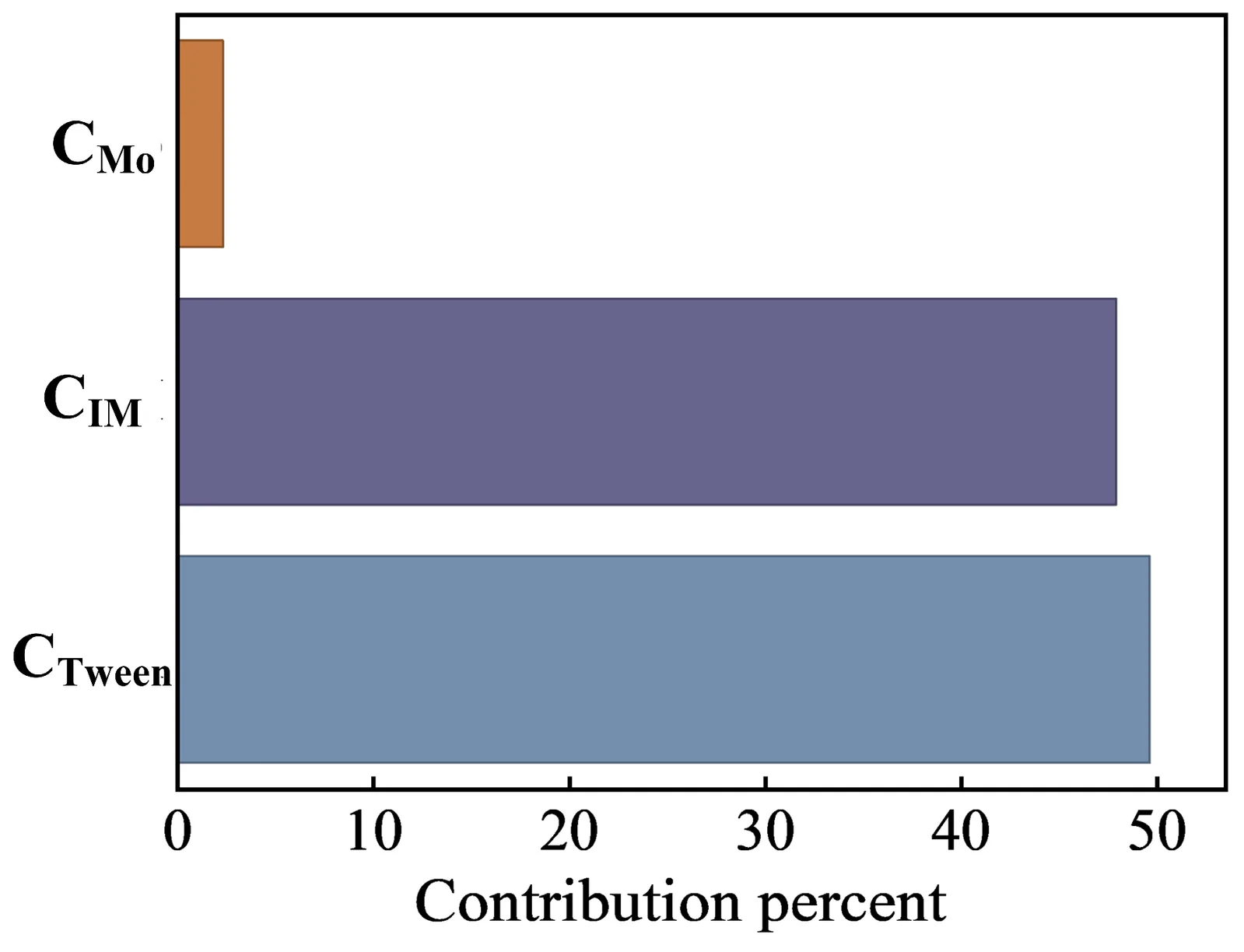
Global SHAP importance of the three formulation variables, summarized by their mean absolute attributions. The normalized shares are 49.7% for *C*_Tween_, 48.0% for *C*_IM_ and 2.3% for *C*_Mo_.

Global SHAP analysis identified *C*_Tween_ and *C*_IM_ as the dominant predictors, with normalized mean absolute attributions of 49.7% and 48.0%, respectively (Figure 10). *C*_Mo_ accounted for the remaining 2.3%.

The normalized SHAP percentages describe the relative magnitudes of model attribution under the selected explanation procedure, rather than the fractions of corrosion protection supplied by the individual ingredients [22,23]. Because the three concentrations are coupled by the fixed total dosage, these percentages do not identify the effect of increasing one component while maintaining all other conditions unchanged. The ranking is therefore interpreted as a model-level description of the concentration predictors, rather than a quantitative hierarchy of chemical efficacy.

The existing electrochemical comparisons provide complementary evidence of composition-dependent behavior at a common total dosage. The formulations containing *C*_IM_/*C*_Mo_/*C*_Tween_ concentrations of 94/33/23, 48/24/78 and 47/75/28 mg L^−1^ all satisfy the 150 mg L^−1^ total-dose constraint. Their different polarization and impedance responses demonstrate the relevance of mixture composition. Notably, the formulation containing 78 mg L^−1^ Tween 80 did not exhibit the largest impedance response among these examples. Thus, the high Tween 80 attribution should not be interpreted as evidence that increasing its concentration necessarily improves protection. Because multiple component concentrations differ between these formulations, the comparisons describe mixture-level behavior rather than isolated component effects.

Similarly, the 2.3% attribution for *C*_Mo_ does not imply that sodium molybdate has negligible mechanistic importance. A small model attribution does not quantify the protective contribution that would be lost if the component were removed. Previous studies indicate that the behavior of organic–molybdate inhibitors depends on mixture composition and solution chemistry [10,11]. In the present study, the surface Mo signal supports the presence of molybdate-associated species at the interface, but does not by itself quantify their protective contribution or establish a specific mechanism. The SHAP results and surface observations are therefore treated as complementary evidence addressing different aspects of the system.

### 3.6. Chemical Interpretation and Comparison with Related Systems

**Figure 11:**
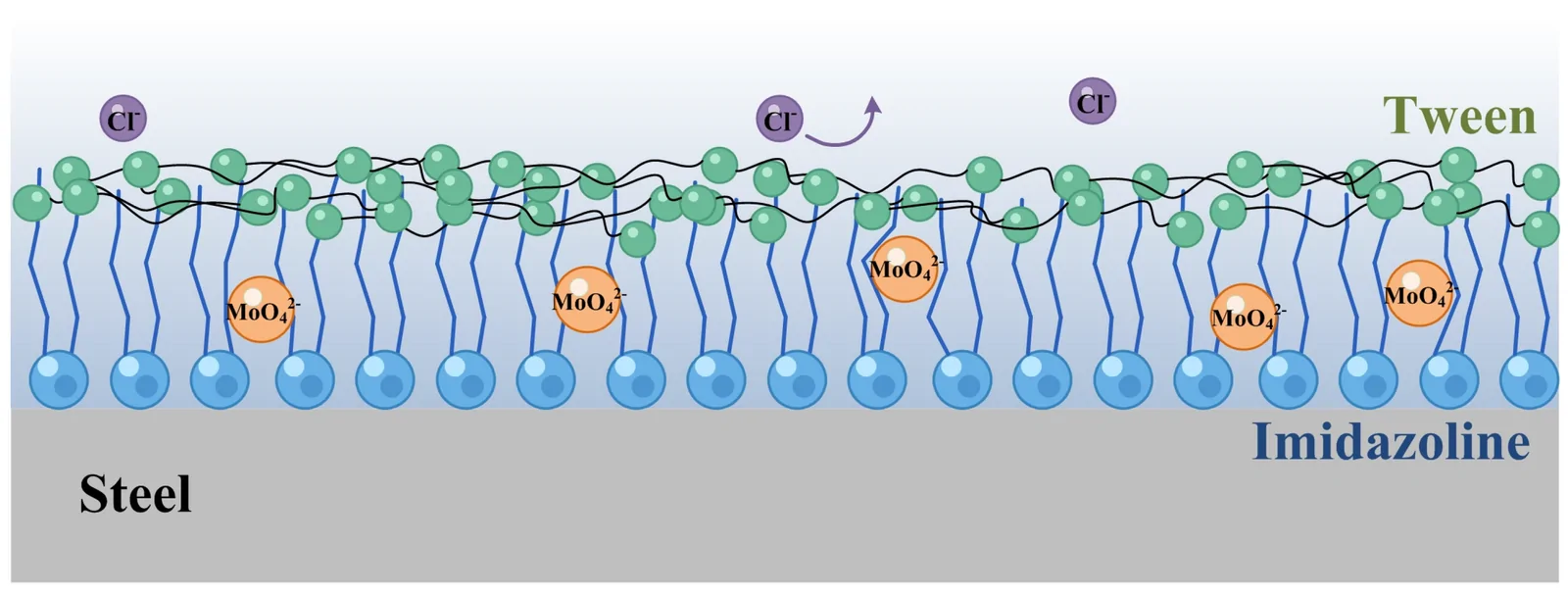
Proposed interfacial protection by IM, molybdate-associated species, and Tween 80. The schematic illustrates a possible combination of organic adsorption, surfactant-mediated organization and inorganic reinforcement.

The experimental observations and the proposed chemical interpretation address different levels of evidence. Polarization and EIS characterize the interfacial electrochemical response, SEM reveals the surface morphology after exposure, and EDS identifies elemental distributions in the examined region. Together, these results are consistent with improved interfacial protection, but do not resolve the chemical states of Mo and Fe or the bonding and molecular organization of adsorbed organic species. Accordingly, Figure 11 presents a plausible interfacial interpretation, with adsorption and molybdate-assisted film formation discussed as hypotheses supported by the combined observations and the relevant literature. IM-derived species may adsorb at the steel–solution interface through their polar groups, while hydrophobic segments reduce access of aggressive solution species [5,6]. Controlled-release work on oleate imidazoline also emphasizes that the availability of the organic inhibitor can influence protection in NaCl solution [7]. In the present mixtures, the strong *C*_IM_ attribution is consistent with sensitivity to organic-inhibitor proportion, while the adsorption geometry remains a proposed molecular interpretation.

Sodium molybdate may reinforce this organic interface through association with surface inorganic species or interactions within the mixed layer. The imidazoline–molybdate–SDBS system provides a closely related example of improved protection on mild steel in NaCl solution [9]. Lin and Zuo also linked lignosulfonate–molybdate inhibition to a combined surface film in an alkaline, chloride-containing concrete pore solution [11]. Although the electrolytes and organic components differ, both studies support considering molybdate as part of a multicomponent protective interface. Here, the Mo signal and increased resistance are consistent with that role, without requiring assignment of a specific Mo-containing phase.

Tween 80 can influence the balance between organic-inhibitor delivery, adsorption and aggregation. Its hydrophilic and hydrophobic moieties make dispersion and interfacial organization plausible contributors to the observed formulation dependence. Studies of Tween 80-containing blends link composition to aggregation and interfacial tension, while nonionic CMEA has shown adsorption-mediated steel protection in an acidic electrolyte [12,13]. These findings provide complementary precedents for a surfactant contribution to the protective interface. In the present system, the high *C*_Tween_ attribution supports the importance of this formulation variable, but does not identify a particular aggregation transition.

The comparison with the imidazoline–molybdate–SDBS formulation is especially informative because both studies involve mild steel in 3.5 wt.% NaCl solution [9]. Replacing anionic SDBS with nonionic Tween 80 changes the potential electrostatic interactions and the balance of adsorption and dispersion within the mixture. The earlier formulation therefore provides a chemical reference, while its molecular interaction scheme should not be assumed to apply unchanged. The transferable principle is that the organic inhibitor, inorganic additive and surfactant must be considered together when interpreting interfacial resistance.

Composition-dependent cooperation is also evident in other inhibitor families. Tang et al. found that the molar ratio of pyridine derivatives to sulfur-containing compounds influenced carbon-steel protection in CO_2_-saturated 3.5 wt.% NaCl solution [34]. Their experimental and computational analysis related this behavior to complementary adsorption within a mixed film. This comparison highlights how a formulation can depend on the organization of unlike components at the interface. A complementary perspective is provided by Temiz et al. [35], who reported improved mechanical performance but diminished corrosion resistance after pack boriding of Fe-20Cr-5Al in sulfuric acid. They associated this behavior with altered passivation and micro-galvanic interactions involving the heterogeneous boride layer and its defects. Their findings illustrate that surface-layer formation and mechanical improvement alone do not establish corrosion protection. The systems nevertheless differ fundamentally: pack boriding produces a diffusion-modified layer through high-temperature treatment, whereas the present study concerns interfacial material associated with solution-phase inhibitor exposure of mild steel in NaCl. The comparison concerns the relationship between layer properties and electrochemical behavior. The relationship between processing, microstructure and corrosion behavior provides a broader context for these observations. Studies of MnAlCuFeTi [36] and AlFeCuTiNi [37] high-entropy materials reported changes in microstructural characteristics and electrochemical behavior with mechanical alloying time and sintering temperature. These results illustrate that corrosion performance depends on the material and its processing state, and that microstructural refinement does not imply a universally improved corrosion response. Such processing-induced changes in the metallic substrate differ from the adsorption and interfacial-film processes proposed for solution-phase inhibitors. In the present study, inhibitor composition was varied within a common steel and surface-preparation protocol; processing conditions and substrate microstructure were not investigated as independent variables.

Accordingly, the current machine learning framework describes the relationship between inhibitor composition and *R*_total_ for the investigated steel–electrolyte system. Its predictors comprise the three inhibitor concentrations, without explicit descriptors of grain structure, phase distribution or processing history. The resulting composition–response relationships therefore support formulation assessment under the studied conditions, while extension to other alloys or processing states would require substrate-specific validation.

The model predicts the EIS-derived total interfacial resistance, *R*_total_ = *R*_p_ + *R*_ct_, rather than corrosion rate or long-term protection. Here, Rp denotes pore- or film-related resistance; conversion of *R*_total_ into corrosion rate would require an appropriate electrochemical relationship and independently justified kinetic parameters [33]. The model is intended for formulation assessment within the sampled composition domain at a total inhibitor dosage of 150 mg L^−1^ under the investigated conditions. Application to other inhibitor concentrations, NaCl concentrations, exposure durations, temperatures or steel compositions would require further validation. Independently prepared formulations and external experimental datasets would provide additional evidence of practical transferability.

## 4. Conclusions

An interpretable composition–resistance model was established for an imidazoline–sodium molybdate–Tween 80 inhibitor in 3.5 wt.% NaCl solution. The framework integrates formulation sampling, electrochemical and surface characterization, Bayesian-optimized stacking and SHAP analysis. The main findings are summarized below.

1. The 90 formulations exhibited *R*_total_ values ranging from 940.21 to 1358.51 Ω·cm^2^. This response combines pore- or film-related resistance with charge-transfer resistance and provides a consistent descriptor for comparing the investigated mixtures.
2. Stacking achieved the most favorable reported point estimates under the implemented evaluation protocol, with R^2^ = 0.9036, MSE = 969.46 (Ω·cm^2^)^2^ and MAPE = 2.22%. Its MSE was approximately 5.4% lower than that of SVR, while Lasso also captured much of the response variation. These comparisons describe numerical differences within the present evaluation rather than statistically established superiority.
3. Tween 80 and IM dominated the normalized mean absolute SHAP attribution, contributing 49.7% and 48.0%, respectively, compared with 2.3% for sodium molybdate. These values identify influential composition variables in the fitted model and support joint consideration of the two organic components during formulation adjustment.
4. Polarization, EIS and surface observations were consistent with improved interfacial protection under the investigated conditions. Organic adsorption, surfactant-mediated organization, and molybdate-assisted reinforcement provide plausible explanations for this behavior, while the specific chemical states and molecular structure of the interfacial material remain to be established.

The framework connects measured interfacial resistance with interpretable formulation trends, supporting prioritization of mixtures within the studied domain. Its practical value lies in combining predictive performance with chemically informed interpretation while retaining the distinction between model attribution and molecular mechanism.

## Figures and Tables

**Table 1:** Chemical composition of mild steel (wt.%).

Element	C	Si	Mn	S	P	Fe
Content	0.20	0.30	0.70	0.042	0.042	Balance

**Table 2:** Summary statistics for the 90 formulation records and the target response.

Variable	*n*	Mean	SD	Minimum	Maximum
*C*_IM_	90	59.03	18.26	20.00	95.00
*C*_Mo_	90	46.72	19.89	10.00	78.00
*C*_Tween_	90	44.24	18.99	10.00	78.00
*R*_total_	90	1139.18	100.84	940.21	1358.51

Concentrations are expressed in mg·L^−1^ and *R*_total_ in Ω·cm^2^. SD denotes the standard deviation across the 90 formulation records.

**Table 3:** Training and test performance of the evaluated regression models.

Model	Training R^2^	Training MSE	Training MAPE (%)	Test R^2^	Test MSE	Test MAPE (%)
Stacking	0.9300	704.19	1.85	0.9036	969.46	2.22
SVR	0.9074	930.85	2.08	0.8981	1024.43	2.24
Lasso	0.8993	1012.75	2.21	0.8938	1068.19	2.28
KNN	0.9327	676.77	1.84	0.8848	1158.32	2.46
CatBoost	0.9916	84.30	0.62	0.8724	1283.50	2.48
LightGBM	0.9213	791.39	1.92	0.8555	1453.00	2.63

## Data Availability

Summary statistics and representative results are presented in this article.
